# Lucanthone Targets Lysosomes to Perturb Glioma Proliferation, Chemoresistance and Stemness, and Slows Tumor Growth *In Vivo*

**DOI:** 10.3389/fonc.2022.852940

**Published:** 2022-04-14

**Authors:** Daniel P. Radin, Gregory Smith, Victoria Moushiaveshi, Alexandra Wolf, Robert Bases, Stella E. Tsirka

**Affiliations:** ^1^ Department of Pharmacological Sciences, Renaissance School of Medicine at Stony Brook University, Stony Brook, NY, United States; ^2^ Stony Brook Medical Scientist Training Program, Renaissance School of Medicine at Stony Brook University, Stony Brook, NY, United States; ^3^ Department of Radiology, Montefiore Medical Center, New York City, NY, United States; ^4^ Department of Radiation Oncology, Montefiore Medical Center, New York City, NY, United States

**Keywords:** autophagy, glioma, cancer stem cell, angiogenesis, hypoxia, lucanthone

## Abstract

Glioblastoma is the most common and aggressive primary brain tumor in adults. Median survival time remains at 16-20 months despite multimodal treatment with surgical resection, radiation, temozolomide and tumor-treating fields therapy. After genotoxic stress glioma cells initiate cytoprotective autophagy, which contributes to treatment resistance, limiting the efficacy of these therapies and providing an avenue for glioma recurrence. Antagonism of autophagy steps has recently gained attention as it may enhance the efficacy of classical chemotherapies and newer immune-stimulating therapies. The modulation of autophagy in the clinic is limited by the low potency of common autophagy inhibitors and the inability of newer ones to cross the blood-brain barrier. Herein, we leverage lucanthone, an anti-schistosomal agent which crosses the blood-brain barrier and was recently reported to act as an autophagy inhibitor in breast cancer cells. Our studies show that lucanthone was toxic to glioma cells by inhibiting autophagy. It enhanced anti-glioma temozolomide (TMZ) efficacy at sub-cytotoxic concentrations, and suppressed the growth of stem-like glioma cells and temozolomide-resistant glioma stem cells. *In vivo* lucanthone slowed tumor growth: reduced numbers of Olig2^+^ glioma cells, normalized tumor vasculature, and reduced tumor hypoxia. We propose that lucanthone may serve to perturb a mechanism of temozolomide resistance and allow for successful treatment of TMZ-resistant glioblastoma.

## Introduction

Gliomas are primary cancers of the central nervous system (CNS) (1). Among them, Glioblastoma (GBM), the highest grade and most aggressive glioma in adults, is the most commonly diagnosed and aggressive glioma in adults (1). The standard of care therapy for GBM consists of maximum safe surgical resection followed by fractional radiation, chemotherapy with the alkylating agent temozolomide (TMZ) and adjuvant treatment with tumor-treating fields (2). Median survival time after diagnosis is approximately 16-20 months (2). As GBM is highly invasive, resection is typically incomplete, which accounts for rapid recurrence and contributes to the universal lethality of this malignancy.

During disease progression, patients often experience comorbidities including pharmacoresistant seizures, headaches, sleep disturbances and neurological deficits in addition to the side effects of radiation and chemotherapy (1), pointing to a great need for new treatment regimens. The search for treatment modalities is complicated by the fact that large molecules cannot pass efficiently through the blood brain barrier, so reagents demonstrating *in vitro* efficacy may not be useful *in vivo* because they never reach the brain. Gliomas are comprised of multiple cell populations including glioma cancer stem cells (GSC), pericytes, infiltrating bone-marrow derived macrophages (BMDM) and microglia (3–5). In glioma, BMDMs and microglia accumulate in tumor tissue attracted by chemokines, such as CSF1 and CCL2, secreted by tumor cells (6, 7) and constitute the glioma-associated macrophages/microglia (GAM). GAM promote glioma cell survival, neoangiogenesis and foster an immunosuppressive tumor microenvironment (TME) (3, 4, 6, 7). These processes constitute targets for novel methodologies to manage GBM.

Accumulating reports in the literature suggest that induction of autophagy in glioma cells promotes resistance to standard of care therapies and survival in hypoxia (8–11). Autophagic induction in tumor-associated pericytes and GAM fosters an immunosuppressive TME (5, 12). In addition induction of autophagy has been reported to limit the oncolytic capacity of cytotoxic T-lymphocytes (CTL) in other tumors (13, 14). Based on this evidence, we hypothesized that inhibiting autophagy may not only augment the efficacy of standard of care therapies, but may also reverse the immunosuppressive TME.

Lucanthone (marketed as Miracil D) is an anti-schistosome agent (15–20). It inhibits topoisomerase II and AP endonuclease 1 (APE1) (21–24). Lucanthone has shown efficacy against solid tumors when paired with ionizing radiation (25). It can cross the blood brain barrier and was shown to induce regression of breast cancer metastases (26) synergizing with TMZ against breast tumor cells *in vitro* (23). Inhibition of autophagy and lysosomal membrane permeabilization (27), may explain lucanthone’s interaction with TMZ and radiation (23, 26). Of particular note, lysosomal membrane permeabilization by chloroquine resulted in repolarization of tumor-associated macrophages from an immune-suppressive/pro-tumor ‘M2-like’ to an immune-promoting/anti-tumor ‘M1-like’ phenotype (28). This phenotypic shift was denoted by a marked increase in pro-inflammatory markers (IFN-γ, TNF-α, CD86, iNOS), a decrease in the expression of anti-inflammatory proteins (IL-10, Arg1) and the induction of anti-tumor T-cell immunity (28). These data suggest that lucanthone’s various mechanistic engagements may potentially serve to target multiple processes that support tumor growth, be it directly on the glioma cells, or indirectly on the GAM, thus augmenting the efficacy of TMZ and radiation, and modulating GAMs to exert anti-tumor effects and promote immune-mediated tumor rejection.

In this study we show that lucanthone targeted glioma cells at clinically relevant concentrations by blocking autophagy. Further, we show that this drug synergized with TMZ and preferentially targeted glioma stem-like cells *in vitro* and slowed tumor growth *in vivo*. Lucanthone normalized tumor vasculature, reduced hypoxia and increased cytotoxic T cell infiltration into the tumor core. All these events highlight the potential robust efficacy of this drug against TMZ-resistant gliomas, which are not normally conducive to chemotherapeutic treatment.

## Materials and Methods

### Cell Culture

GL261 cells expressing luciferase (GLUC2) were obtained from the lab of Dr. Michael Lim. They are derived from a chemically induced astrocytoma in C57BL/6 mice (29). KR158 cells were obtained from the labs of Drs. Tyler Jacks and Behnam Badie, and are derived from genetically engineered Nf1/Tp53 mutants (30). Cells were maintained in DMEM, 10% serum, 1% antibiotic, 1% sodium pyruvate and incubated at 37°C with 5% CO_2_. bEND.3 cells were cultured in DMEM with serum as above. Primary patient-derived human glioma cells (GBM43) which carries Nf1 and Tp53 mutations were obtained from Dr. Jann Sarkaria at the Mayo Clinic from the xenograft cell line panel. To enrich for glioma stem-like cells (GSC) in GLUC2, KR158 and GBM43 cells, serum was reduced step-wise over a week as described previously (31). GSC were cultured in serum-free DMEM medium containing F12 supplement along with pyruvate, antibiotics, N2 supplement, EGF, FGF and heparin (31).

### Crystal Violet Studies

For single lucanthone treatment studies, GLUC2 and KR158 cells were plated at a density of 2,000 and 1,000 cells per well, respectively, in a 6-well plate and allowed to adhere overnight. They were then treated with 10 μM Lucanthone every 4 days for 12 days. On day 13, media were aspirated, and cells were fixed with 4% PFA for 10 minutes. Cells were then treated with 0.5% crystal violet solution for 20 minutes. Plates were washed and photographed.

For dual treatment studies (lucanthone and TMZ), GLUC2 and KR158 cells were plated at a density of 2,500 and 1,000 cells per well in a 12-well plate and allowed to adhere overnight. Cells were then treated with medium, TMZ, lucanthone, or a combination for 4 days. The media were aspirated, and the cells were washed with PBS once and incubated with standard medium for 3 days. The cells were fixed with PFA and treated with 0.5% crystal violet solution as above and photographed. Then lysis solution of 10% SDS in dH_2_O was added to the plates overnight. To quantify relative crystal violet intensity, the absorbance of the crystal violet-containing supernatant was read under a spectrophotometer at 590 nm with a reference wavelength of 670 nm. Data are graphed as percent of control (medium only-treated cells).

### MTT Assay

Cells were plated in a 96-well plate and incubated overnight. Adherent tumor cells (2D cultures) were treated with lucanthone for 3 days and then subject to the MTT protocol as per manufacturer’s instructions (Promega). GSC (3D cultures) were treated with lucanthone for 5 days, as this allowed sufficient time for spheroids to grow in culture. Prior to addition of the MTT reagent, plates were imaged under confocal microscopy with the addition of Calcein-AM and Ethidium homodimer to mark live cells and dead cells, respectively.

### Acridine Orange Stain

GLUC2, KR158 and GBM43 cells were plated on glass-bottom 35mm plates overnight. They were then treated with medium or lucanthone for 48 hours. The cells were treated with 5μg/ml acridine orange for 15 minutes. Plates were washed with PBS 3x and then incubated in complete medium. Plates were then imaged for acidic vesicle accumulation (525/590nm) under confocal microscopy, according to manufacturer’s instructions (Cayman chemical).

### Immunocytochemistry

For immunocytochemical analysis, GLUC2, KR158 and GBM43 cells were plated on glass coverslips overnight. Cells were treated with medium or lucanthone for 48 hours. The medium was aspirated and cells were fixed with 4% PFA for 10 minutes. Plates were then washed 3x with 0.3% TX-100 in PBS and wells were blocked with 3% normal goat serum/0.3% TX-100 in PBS for 1 hour. Cells were stained with primary antibodies overnight (LC3, Ki67, Nestin, Olig2, SOX2, CD133, p62, Cathepsin D, γH2AX). The primary antibody was removed, and cells were again washed 3x with 0.3% TX-100 in PBS after which time cells were incubated with fluorescent secondary antibodies for an hour at room temperature. Cells were then washed 3x with PBS, counterstained with DAPI and imaged under confocal microscopy. GSC were induced to adhere to glass slides by precoating glass slides with Geltrex for an hour.

### Western Blot

Immunoblotting was done as described previously (3). Briefly, cells were lysed in 50mM Tris-HCl (pH 7.4) with 1% Nonidet P-40, 0.25% sodium deoxycholate, 150mM NaCl, 1% SDS and 1mM sodium orthovanadate. Proteins were denatured by boiling with treatment with BME. Proteins were run on SDS-page gels, transferred to PVDF membranes (Immobilon; Millipore). Membranes were washed with Tris-buffered saline with 0.1% Tween 20 and blocked in a 5% non-fat dry milk powder for 1 hour. Membranes were then probed for LC3 (1:1000), p62 (1:1000), Olig2 (1:1000), SOX2 (1:1000) and B-Actin (1:2000; sigma Aldrich). Membranes were rinsed in TBS-T, probed with associated HRP-conjugated secondary antibodies and exposed to Pierce ECL substrate for 1 minute (Thermo Fisher Scientific) after which x-ray films were developed from membranes.

### RNA Isolation and Quantitative RT-PCR

To prepare RNA, GLUC2 spheroids were spun down and lysed with Trizol and processed using the manufacturers protocol. To obtain cDNA, one microgram of RNA was reverse transcribed on a Veriti thermocycler using the High Capacity cDNA Reverse Transcription Kit. Amplification was performed on a StepOnePlus real-time PCR machine using a SYBR green kit (Applied biosystems). Primer sequences are as follows: GAPDH forward, 5′-GCACAGTCAAGGCCGAGAAT-3′; GAPDH reverse, 5′-GCCTTCTCCATGGTGGTGGA-3′; Olig2 forward, 5′- CAAATCTAATTCACATTCGGAAGGTTG -3′; Olig2 reverse, 5′- GACGATGGGCGACTAGACACC -3′. GAPDH was used as an internal control.

### Animals

C57Bl6 mice were bred under maximum isolation on a 12:12 hour light:dark cycle with food *ad libitum*.

### Murine Glioma Model

Gliomas were established in 3-4 month old male and female mice as described previously (3, 4, 32). GLUC2 GSC were dissociated with accutase and counted. Mice were anesthetized with 20mg/kg avertin, a midline incision was made in the scalp, the skin retracted and a small burr hole was drilled in the skull at the following stereotactic coordinates from bregma: -1mm anteroposterior and +2 mediolateral. 1x10^5^ GLUC2 GSC resuspended in PBS were injected over a period of 2 minutes at a depth of 3mm. At the end of the injection, the needle was kept in the injection site for a further 3 minutes. After needle removal, the incision was sutured and mice were placed on a heating pad until they fully recovered from anesthesia. During the disease course if mice were found to have lost more than 15% of their initial body weight, they were euthanized. All animal procedures were approved by the Stony Brook University Institutional Animal Care and Use Committee.

### *In Vivo* Luciferase Imaging

GSC engraftment was visualized using the IVIS spectrum *in vivo* imaging system 7 days after inoculation and again on days 14 and 21. Briefly, mice were anesthetized using continuous isofluorane exposure. Their scalps were shaved. Mice were injected i.p. with 150mg/kg D-Luciferin, carefully placed in the IVIS spectrum machine and imaged every 3-4 minutes for 40 minutes. Relative signal was quantified by a researcher blinded to the treatment, and luminescence ratios of day 21 to day 7 were calculated to approximate disease progression throughout the course of treatment.

### Lucanthone Treatment *In Vivo*

Lucanthone was supplied by Dr. Robert Bases. Lucanthone was solubilized in 10% DMSO, 40% HPCD in PBS. After confirming the presence of gliomas on day 7, mice were randomly divided to control and treatment groups, and treated with either saline or 50mg/kg Lucanthone i.p. every day from day 7 to day 20. On day 21, tumors were visualized by bioluminescent imaging, as above.

### Immunohistochemistry

Mice were anesthetized with 20mg/kg avertin and transcardially perfused with 30ml PBS followed by 30ml 4% PFA in PBS. Brains were removed and post-fixed in 4% PFA in PBS overnight. They were dehydrated for 48 hours in 30% w/v sucrose in PBS. Brains were then embedded in optimal cutting temperature compound (OCT, Tissue-Tek) and 20μm coronal sections throughout the entire tumor were taken on a Leica cryostat (Nusslock, Germany) and collected on Superfrost plus microscope slides. To determine tumor volume, serial sections were taken from each animal and subjected to hematoxylin and eosin stain. Tumor volume was calculated as tumor area x 20 μm thickness, x number of slides (33).

For immunohistochemical analysis, slides were brought to room temperature, washed 3x with 0.3% TX-100 in PBS and then blocked with 1% BSA/0.3% TX-100 in PBS for 1 hour. Slides were incubated overnight with appropriate primary antibodies (**Supplementary Table 1**). The primary antibody was removed and slides were washed 3x 0.3% TX-100 in PBS and incubated with appropriate secondary antibodies for 1 hour. Slides were washed 3x with PBS, and counterstained with DAPI. Immunoreactivity was visualized by confocal imaging using the Leica SP8-x system, with white light and argon lasers.

### Statistical Analysis

Data comparing two population means with a normal distribution were analyzed using Student’s t-test. Data with non-normal distributions were analyzed using a Mann-Whitney test. Differences in cumulative distributions were assessed with the Kolomogorov-Smirnov test. To assess for synergistic interactions, King’s synergy test was used (34–36). Blood vessel circularity was calculated using the equation Circularity=4*π*(area/(perimeter^2^)). Alpha value was set at 0.05 prior to starting experiments. Power analysis was used to determine the appropriate number of animals used in each experiment. Experiments were replicated with the two tumor lines. Statistical analysis was performed using Graphpad Prism (Graphpad Software Inc, La Jolla, CA).

## Results

### Lucanthone Targets Lysosomes and Inhibits Autophagy

**Figure 1:**
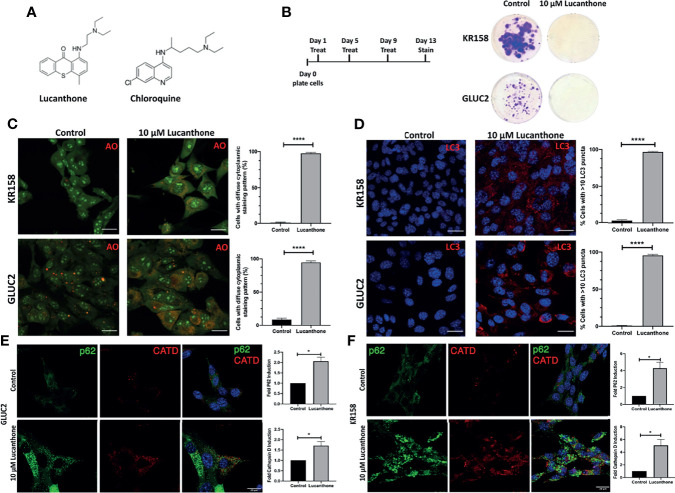
Lucanthone compromises glioma cell growth. **(A)** Chemical structures of lucanthone and chloroquine. **(B)** Effects of long-term treatment of KR158 and GLUC2 cultures with 10 μM lucanthone on glioma cell proliferation. **(C)** Acridine orange (AO) marks lysosomes as punctae staining after 48 hour of lucanthone treatment. **(D)** LC3 marks autophagosome punctae levels after 48 hour treatment with lucanthone. **(E, F)** Effect of lucanthone on P62 and Cathepsin D levels in GLUC2 and KR158 cells, respectively. Scale bar = 30 μm. Bars are mean +/- SEM. N= 3-4 independent experiments. *p < 0.05, ****p < 0.0001, student’s t-test.

To examine whether lucanthone affects the growth of the two murine glioma cell lines GLUC2 and KR158, lucanthone (**Figure 1A**) was added to glioma cultures at 10 μM every 4 days for 2 weeks (**Figure 1B**), which reflects concentrations observed in the serum of patients (26). The proliferation of both cell lines was hindered. To investigate whether the possible mechanism by which lucanthone acts on glioma cells engaged autophagy, we treated glioma cells with lucanthone for 48 hours, and then stained them with acridine orange, which accumulates in acidic vacuolar organelles and shifts from green to red fluorescence (37). In control conditions, only few lysosomes were present in the cell lines. After treatment with lucanthone, cultures in both cell lines exhibited a remarkable diffuse cytoplasmic staining of dilated lysosomes (**Figure 1C**) with a corresponding increase in LC3 punctae (**Figure 1D**). These data parallel what has been observed after treatment with chloroquine in other tumor types (37) and suggest that lucanthone targets lysosomes and affects autophagic function at clinically relevant concentrations.

We also assessed the levels of the autophagy cargo receptor p62 and Cathepsin D. P62 accumulates in cells in which autophagy has been functionally inhibited and Cathepsin D is a lysosomal aspartyl protease (27). Our data demonstrate that after 48 hours of lucanthone treatment, P62 and Cathepsin D increase in both glioma cell lines, though we note a higher relative increase of both proteins in KR158 cells (**Figures 1E, F**). These findings illustrate lucanthone’s ability to inhibit autophagy at clinically relevant concentrations.

To examine whether lucanthone exerts its functions by acting as an inhibitor of topoisomerase 2 or APE1, we assessed the extent to which lucanthone induced DNA damage in glioma cell lines. To that end, GLUC2 and KR158 cells were treated with lucanthone for 48 hours, after which levels of γH2AX, a DNA damage marker, were assessed (38). As a positive control, glioma cells were also treated with the FDA-approved topoisomerase 2 inhibitor etoposide. While etoposide produced a marked increase in γH2AX intensity, lucanthone only produced a minimal effect, indicating that it is exerting its effect most likely *via* autophagy inhibition. When the levels of cleaved caspase-3 were evaluated, only minimal induction of cleaved caspase-3 in GLUC2 and KR158 spheroids treated with 10 μM lucanthone for 48 hours were observed, indicating that lucanthone may not be inducing apoptosis in these glioma cell lines (**Figure S1**), as was shown for another autophagy inhibitor, thymoquinone, which induces cathepsin-mediated, but caspase-independent cell death (39).

### Lucanthone Interacts With Temozolomide

**Figure 2:**
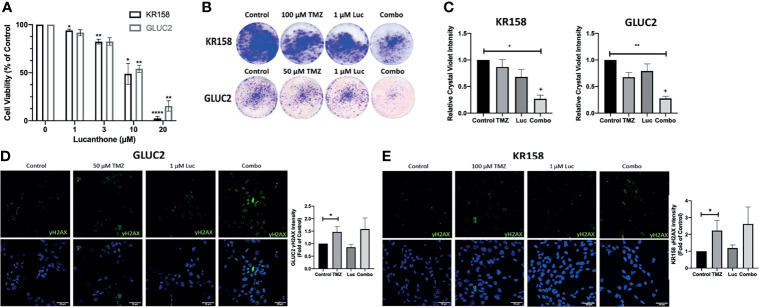
Interaction between lucanthone and temozolomide. **(A)** KR158 and GLUC2 cells were treated with Lucanthone for 72 hours, after which an MTT assay was performed. Bars are mean +/- SEM, N=3-7 independent experiment. ANOVA p<0.0001. *p < 0.05, **p < 0.01, ****p < 0.0001, Dunnett’s multiple comparison test to control-treated cells. **(B, C)** KR158 and GLUC2 cells were treated with lucanthone, TMZ, or the combination for 4 days and then allowed to recover in drug-free medium for 3 days. The cells were PFA-fixed and stained with crystal violet. Crystal violet-stained cells were then lysed and relative absorbance was measured to approximate culture viability. Representative wells are shown in **(B)**. **(C)** Quantification of crystal-violet stained cultures. Bars are mean +/- SEM, N=3-4 independent experiments. *p < 0.05, **p < 0.01, Dunnett’s multiple comparison test to control-treated cells. +p < 0.05, King’s synergy test, demonstrating significant interactions between lucanthone and TMZ in both cell lines. **(D)** Representative micrographs of γH2AX stained GLUC2 cells and quantification of γH2AX intensity per number of cells in the field of view in experiments where the GLUC2 cells were incubated with TMZ, or the combination of lucanthone and TMZ. **(E)** Representative micrographs of γH2AX stained KR158 cells. Quantification of γH2AX intensity per number of cells in the field of view in experiments where the KR158 cells were incubated with TMZ, or the combination of lucanthone and TMZ. Bars are mean +/- SEM, N=4 independent experiments. *p < 0.05, Mann-Whitney test.

The interaction between lucanthone and TMZ was investigated by performing combination studies *in vitro*. First, we performed an MTT assay to determine minimally effective concentrations of lucanthone in both cell lines. Lucanthone exerted a dose-dependent reduction in cell viability, with an IC50 of approximately 11-13 μM (**Figure 2B**). Two-way ANOVA illustrated that both cell lines were similarly sensitive to lucanthone, implying that this drug may be useful regardless of driver mutations. These data also pointed towards the use of 1 μM lucanthone for the combination studies, since this concentration exerted minimal effects alone on both cell lines.

It has been reported that GL261 and KR158 cells exhibit striking resistance to TMZ *in vitro* (40, 41). Therefore, we treated GL261 and KR158 cells with control medium, either drug alone, or both drugs for 4 days, and then allowed cultures to recover for 3 days before analysis. In this extended treatment format, 1 μM lucanthone alone, or 50 μM TMZ or 100 μM TMZ produced only a modest effect on GL261 and KR158 cells (**Figures 2B, C** **)**. However, crystal violet intensity was markedly decreased when cells were treated with a combination of lucanthone and TMZ (**Figures 2B, C**, p<0.05, King’s synergy test). Our data, in agreement with previous studies on breast tumor cells (23), suggest that even lower doses of lucanthone may be useful when paired with standard of care therapies to slow glioma progression.

To understand why lucanthone may augment the anti-tumor effects of TMZ, we tested for changes in the levels of γH2AX, a marker of DNA damage, in both cell lines. After 48 hours, changes in γH2AX intensity were evident in cultures treated with TMZ, but not in those treated with lucanthone (**Figures 2D-F** **)**. Cultures treated with both drugs exhibited slightly increased levels of γH2AX compared to cultures treated with TMZ alone, but this increase did not become statistically significant.

### Lucanthone Targets Glioma Cancer Stem Cells and Overcomes Acquired Temozolomide Resistance

Cancer stem cells are defined as progenitor-like tumor cells that repopulate the tumor after what is considered “successful” treatment, driving tumor recurrence and fatality. It is now accepted that cancer stem cells (termed here GSC) are rapidly dividing (42) and resistant to both TMZ and radiation (43, 44). Recent data reveal that GSC preferentially rely on autophagy for their survival and resistance to TMZ (45, 46). To that end, we enriched for stemness characteristics in GLUC2 and KR158 cell lines (please see Materials and Methods). Both glioma cell lines grew as partially suspended spheroids. 1 week after culturing cells in stemness medium, GLUC2 spheroids stained positive for the stemness markers nestin, SOX2 and Olig2, while KR158 spheroids stained positive for nestin, CD133 and SOX2 (**Figure S2**). Cells staining positive for these markers also stained positive for the proliferation marker Ki67, demonstrating that these cells are indeed actively proliferating. Additionally, western blot analysis indicates that GLUC2 spheroids express higher levels of SOX2 and Olig2, while KR158 spheroids express higher levels of SOX2 than their adherent counterparts (**Figure S2** **).**

**Figure 3:**
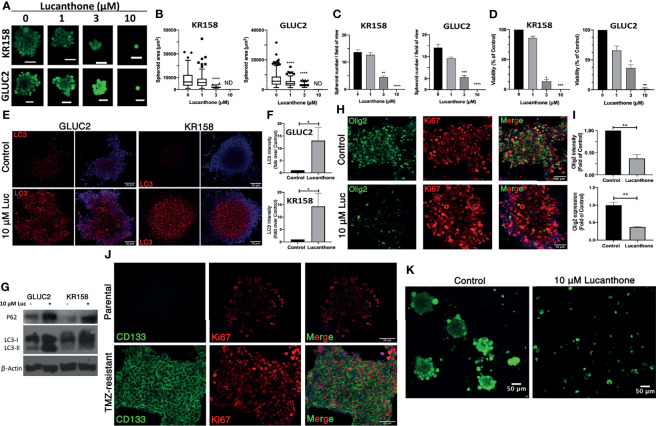
Lucanthone targeted GSC and overcame acquired resistance to temozolomide. GLUC2 and KR158 spheroids were mechanically dissociated, plated overnight and treated with increasing concentrations of lucanthone for 5 days. After treatment, they were stained with Calcein-AM to visualize viable cells. **(A)** Representative images of KR158 and GLUC2 GSC treated with increasing concentrations of Lucanthone for 5 days; **(B)** Spheroid area distribution. ****p < 0.0001, Kolmogorov-Smirnov test comparing distributions to control-treated cultures; ND, Not Detected. **(C)** Spheroid number per field of view; **(D)** Viability of cultures as determined by MTT assay. Bars are mean +/- SEM, N=3-4 independent experiments. *p < 0.05, **p < 0.01, ***p < 0.001, Dunnett’s multiple comparison test to control-treated cells. **(E)** LC3 staining in GLUC2 and KR158 spheroid cultures treated with media or 10 μM lucanthone for 48 hours; **(F)** LC3 intensity measured in the same cultures. *p < 0.05, Mann-Whitney test; **(G)** Olig2 staining in GLUC2 spheroid cultures treated with media (Control) and 10 μM lucanthone-treated for 48 hours; **(H)** Olig2 intensity and mRNA expression in the same cultures. **p < 0.01, t-test. N=3-4 independent experiments; **(I)** Immunoblot analysis of p62 and LC3 in protein extracts from GLUC2 and KR158 spheroids with media or 10 μM lucanthone for 48 hours; **(J)** GLUC2 cells treated with 5 cycles of TMZ stained for the stemness marker CD133 and for the proliferation marker Ki67; **(K)** TMZ-resistant GLUC2 cells treated with media or 10 μM lucanthone for 5 days.

After determining that these cells expressed stemness markers, they were treated with increasing concentrations of lucanthone. Remarkably, doses as low as 3 μM produced a strong oncolytic effect in these GSC. Lucanthone reduced spheroid area in both cell lines (**Figures 3A, B** **)**. Further, treatment with lucanthone in a dose-dependent manner resulted in reduced numbers of spheroids formed in culture and reduced viability of the cultures (**Figures 3C, D** **)**. These data show that lucanthone may preferentially kill cells left behind after treatment with modalities such as TMZ and radiation. Additionally, the IC_50_ of lucanthone was approximately 2μM for KR158 and GLUC2 GSC. This is in contrast to an IC_50_ of 11-13 μM in cells cultured with serum. These data indicate that stem-like glioma cells may be more susceptible to autophagy inhibiting drugs like lucanthone.

To gain mechanistic insight into how lucanthone reduces stemness, we allowed KR158 an GLUC2 GSC to form spheroids for 10 days, then treated spheroids with 10 μM lucanthone for 48 hours and assayed for alterations in levels of LC3and p62. By western blot analysis, we observed that lucanthone increased p62 levels in GLUC2 and KR158 GSC and increased levels of LC3-II as well (**Figures 3E, G**). We also observed increases in LC3 punctae in spheroids by immunocytochemistry and immunoblotting (**Figures 3E-G**). These data illustrate that the drug probably acted in a similar manner to that observed in adherent 2D cultures. It is worthy to note that in control conditions, LC3 punctae were also observed in spheroids, suggesting a higher level of baseline autophagy in GSC and a higher reliance on autophagy in general. In addition to assessing for changes in autophagic flux, we assessed for changes in the levels of stemness markers after treatment. We observed a strong reduction in Olig2 intensity in lucanthone-treated cultures (**Figures 3H, I**), while expression on nestin and SOX2 did not change. Using RT-qPCR we found that lucanthone reduced Olig2 mRNA expression in GLUC2 spheroids by >60% (**Figure 3I**). Minimal changes were also observed in Ki67 in these cultures.

Despite multimodal treatment, the recurrence rate for glioblastoma is ~100%. It has also been proposed that glioma cells change throughout the course of treatment such that the cells that survive treatment are functionally different than the parental tumor (47–49). We tested whether Lucanthone was able to exert oncolytic effects on glioma cells that have been selected for their ability to resist the standard chemotherapy temozolomide, TMZ. To that end, we treated GLUC2 cells with two cycles (48 hours of treatment and 7 days recovery per cycle) of 250 μM TMZ and 3 cycles of 500 μM TMZ. After the selection, we noticed that the surviving cells started forming spheres in serum-containing medium, similar to the ones we observe when culturing these cells in stemness-promoting medium. These spheroids expressed the prototypic stemness gene CD133 whereas parental GLUC2 spheroids did not (**Figure 3J**), suggesting that glioma cells dynamically respond to genotoxic therapy by acquiring stem-like morphology and characteristics (47). Cells selected for TMZ resistance were also less sensitive to TMZ treatment than parental GLUC2 cells (**Figure S3**). In spite of becoming more stem-like, these cultures were still markedly sensitive to 10 μM lucanthone (**Figure 3K**), suggesting that lucanthone could be used to slow the growth of TMZ-resistant malignant glioma cells.

**Figure 4:**
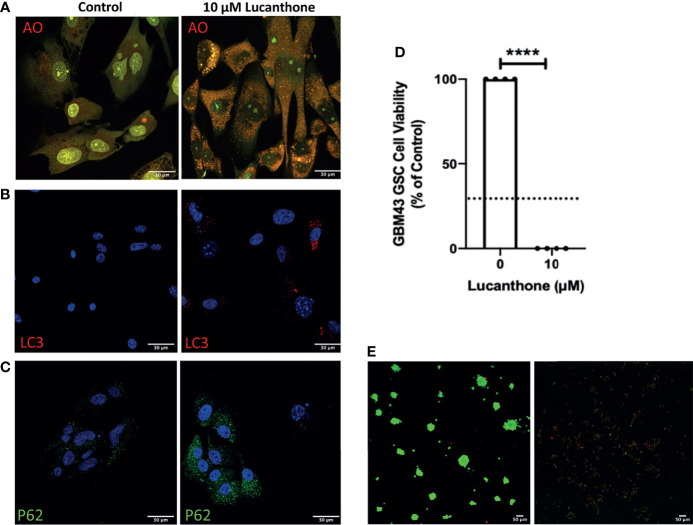
Patient-derived glioma cells are susceptible to lucanthone. **(A)** GBM43 cells were treated with lucanthone and assessed for changes in acridine orange staining, **(B)** LC3 and **(C)** p62 levels. **(D)** GBM43 CSCs were treated with lucanthone for 5 days and then an MTT assay was performed. **(E)** GBM43 GSC were treated with media or lucanthone for 5 days, after which spheroids were visualized by Calcein-AM and Ethidium homodimer staining. Data are representative of 4 independent experiments. ****p < 0.0001, t-test. Dotted line represents culture viability prior to any treatment.

To examine if lucanthone could target human glioma cells as well, we obtained patient-derived glioma cells from the Mayo Clinic (termed GBM43), which bear Tp53 and Nf1 mutations. After treatment with lucanthone, GBM43 cells exhibited a similar acridine orange cytoplasmic staining pattern as seen in GLUC2 and KR158 cells (**Figure 4A**). Additionally, there were modest increases in LC3 and P62 (**Figures 4B, C** **)**, suggesting that autophagy was inhibited in these cells. After enriching for stem-like qualities in these cells, treatment with 10 μM lucanthone drastically reduced cell viability (**Figure 4D**) and completely inhibited spheroid formation in these cultures (**Figure 4E**). Taken together, these data show that lucanthone can be used to inhibit autophagy in mouse and human glioma cells.

### Lucanthone Slows Glioma Growth *In Vivo*

**Figure 5:**
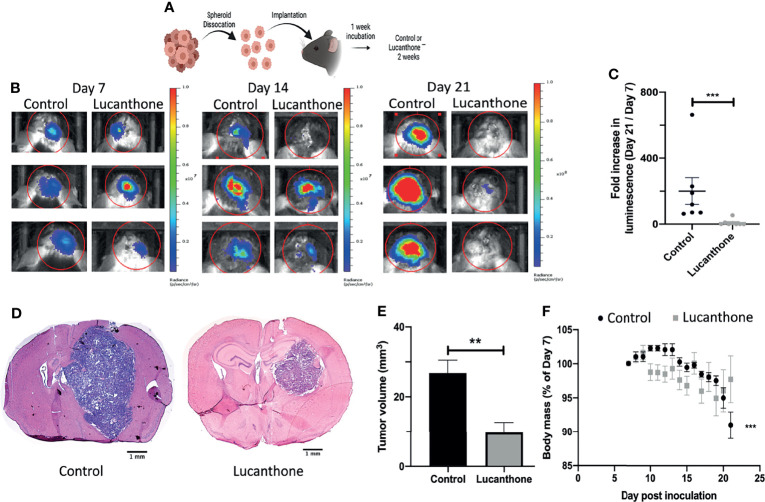
Lucanthone mitigated the growth of dissociated GLUC2 spheroids *in vivo*. **(A)** Treatment scheme used for the study. **(B)** Representative images of *in vivo* luminescent imaging on Days 7, 14 and 21. **(C)** Fold increase in luminescence from day 7 to day 21. ***p < 0.001, Mann-Whitney test. **(D)** Tumor volume of control- and lucanthone-treated animals with representative images shown in **(E)** **p < 0.01, Mann-Whitney test. **(F)** Body mass depicted as a percentage of the start of treatment on day 7. ***p < 0.001, Mann-Whitney test, compared to relative body mass on day 7. Bars are mean +/- SEM, N=7-8 animals.

To assess translational potential, the efficacy of lucanthone was investigated in a mouse model of glioma. GLUC2 GSC were allowed to form spheroids for 10 days in culture. The spheroids were mechanically dissociated and 100,000 GLUC2 cells were implanted in the striatum of mice. Tumors were allowed to form for 7 days. Tumor cell presence was confirmed using IVIS imaging system on day 7, after which mice were segregated into two groups: one group received saline every day until day 21 while the other group received 50mg/kg lucanthone every day until day 21. The animals were imaged on days 14 and 21 (**Figure 5A**). On day 14, 5 of the 7 control mice exhibited a 2-fold increase in luminescence. In contrast, only 1 of 8 lucanthone-treated mice experienced a two-fold increase in luminescence, suggesting that lucanthone mitigated tumor growth between days 7 and 14 (chi-squared test, p<0.05). By day 21, control (saline)-treated glioma-bearing mice experienced a ~200-fold increase in tumor luminescence compared to day 7, whereas lucanthone-treated mice experienced only a 10-fold increase in tumor luminescence (**Figures 5B, C** **)**. Upon histological analysis, the tumors of lucanthone-treated mice were approximately 60% smaller than those of saline-treated animals (**Figures 5D, E** **)**. Moreover, saline-treated mice experienced cachexia (**Figure 5F**), whereas lucanthone-treated mice did not experience significant weight loss throughout the course of treatment (**Figure 5F**).

### Lucanthone Reduces Olig2^+^ Glioma Cells *In Vivo*

**Figure 6:**
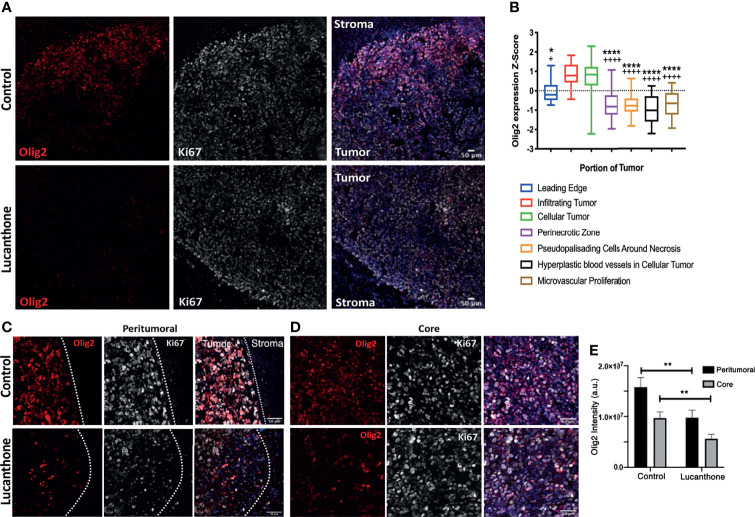
Lucanthone reduced Olig2^+^ positivity in tumors *in vivo*. **(A)** Representative immunohistochemical images of Olig2 and Ki67 in tumors and surrounding stroma in saline- and lucanthone-treated mice. **(B)** Expression of Olig2 in different areas in human glioblastomas adapted from the Ivy Glioblastoma Atlas. ****p < 0.0001 Kruskal-Wallis test, demonstrating significant differences in Olig2 expression among various tumor areas. *p < 0.05, ****p < 0.0001 Dunn’s test, compared to infiltrating tumor. ^+^p < 0.05, ^++++^p < 0.0001, Dunn’s test, compared to cellular tumor. **(C, D)** Olig2 expression in tumor periphery and tumor core in both treatment conditions with intensity quantifications in **(E)** Two-way ANOVA p < 0.05. **p < 0.01, Bonferroni multiple comparison test. Bars are mean +/- SEM, N=4 animals per group.

Standard of care therapies for glioma enrich for tumor stem-like cells, which is thought to play a role in glioma recurrence (43, 44). However, the *in vitro* data described so far suggest that lucanthone may reduce stem-like qualities of glioma cells, rather than solely target non-stem glioma cells. To that end, we interrogated how lucanthone affects glioma stem-like cells *in vivo*. The expression of stemness genes such as Olig2 and SOX2 was assessed in experimental tumors. Initial examination revealed that the density of Olig2^+^ cells was highest near the periphery of the tumor (**Figures 6A, C, D** **)**, though we did observe a significant number of Olig2^+^ cells near the core as well. These data agree with previous findings that Olig2^+^ glioma cells are present at increased numbers near the tumor periphery (50). According to the Ivy Glioblastoma Atlas, an anatomically annotated transcriptional dataset of human glioblastoma tumors (51), Olig2 expression is increased in areas of infiltrating tumor and cellular tumor, and reduced in areas of necrosis and around blood vessels (**Figure 6B**). These findings suggest that, with respect to spatial expression of Olig2, GLUC2 GSC may accurately reflect what is observed in the human disease.

In contrast to the abundant Olig2 expression observed in saline-treated mice, we noted a striking reduction in Olig2 positivity around the periphery of lucanthone-treated tumors and near the core of these tumors. Two-way ANOVA revealed that in both treatment conditions, Olig2 intensity is higher near the tumor border, and that lucanthone resulted in reduction of Olig2 intensity at the tumor periphery and in the tumor core (**Figure 6E**). Ki67 positivity was similar in both treatment conditions. Additionally, SOX2 expression was not significantly different between treatment conditions, which parallels the result when individual spheroids were treated with lucanthone *in vitro*. While lucanthone did not significantly modulate γH2AX *in vitro*, γH2AX positivity was modestly increased *in vivo* in lucanthone-treated tumors (**Figure S4**). Increases in γH2AX were most likely restricted to glioma cells, as most of the cells that exhibited increases in γH2AX were not staining for the GAM marker, F4/80 (**Figure S4**).

### Tumor Microenvironmental Changes Induced by Lucanthone

**Figure 7:**
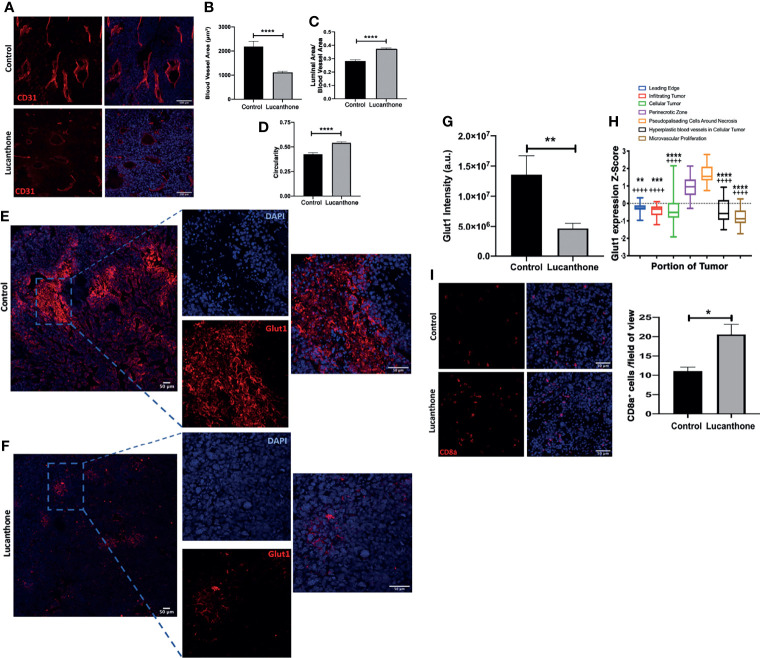
Tumor microenvironmental changes induced by Lucanthone. **(A)** Representative images of blood vessels marked by CD31 of control- and lucanthone-treated tumors. **(B)** Blood vessel area. **(C)** Luminal area/blood vessel area. **(D)** Blood vessel circularity. ****p < 0.0001, Kolmogorov-Smirnov test. Bars are mean +/- SEM, N=4-5 animals per group. **(E, F)** Representative images of Glut1 levels in control- and lucanthone-treated tumors, respectively. **(G)** Quantification of Glut1 intensity in the tumor microenvironment. Bars are mean +/- SEM. N=5 mice **p < 0.01, t-test. **(H)** Glut1 expression in necrotic areas in clinical specimens. Data adapted from the Ivy Glioblastoma Atlas. ****p < 0.0001, Kruskal Wallis test. **p < 0.01, ***p < 0.001, ****p < 0.0001, Dunn’s test, compared to perinecrotic zone, ^++++^p < 0.0001, Dunn’s test, compared to pseudopalisading cells around necrotic areas. **(I)** CD8a^+^ cells in the tumor microenvironment in control- and lucanthone-treated tumors. *p < 0.05, Mann-Whitney test Bars are mean +/- SEM, N=4 animals per group.

In addition to assessing for tumor-cell specific effects of lucanthone *in vivo*, the extent to which other cell types in the tumor microenvironment may have been functionally affected by lucanthone treatment was examined. Previously, evidence has been provided that in addition to directly targeting tumor cells, chloroquine (another autophagy inhibitor) normalized the formation of blood vessels in the tumor microenvironment by directly acting on endothelial cells (52). Chloroquine augmented Notch1 signaling in endothelial cells, and as a consequence, reduced the blood vessel tortuosity and increased blood vessel patency. Because lucanthone and chloroquine exert their effects by a similar mechanism, we hypothesized that lucanthone may also modulate blood vessel formation in developing gliomas. To examine this possibility, tumor sections were stained for CD31, an endothelial cell marker. Blood vessel area, luminal area and overall blood vessel circularity were assessed. Interestingly, large blood vessels were observed in control tumors, though many of them exhibited a small luminal area. Accordingly, there were multiple tortuous blood vessels with minimal circularity. In lucanthone-treated tumors, the blood vessels were smaller, but those blood vessels typically showed an increased luminal area and the blood vessels themselves were more circular, suggesting that lucanthone may indeed be functionally affecting angiogenesis in the tumor microenvironment, potentially by acting directly on endothelial cells (**Figures 7A-D**). CD31 intensity was also diminished in lucanthone-treated tumors (**Figures 7A-D**). To examine if lucanthone acted directly on endothelial cells, we treated bEND.3 cells with lucanthone for 72 hours. Lucanthone exerted a dose-dependent effect on the cells, as at 20μM it significantly reduced bEND.3 cells viability after incubation for 72 hours (**Figure S5**).

To interrogate functional outcomes of normalized tumor vasculature, the extent to which tumors exhibited evidence of hypoxia was assessed. In addition to proteins such as Hif1a/Hif2a, there are multiple other proteins induced in areas of tumor hypoxia, including Carbonic Anhydrase IX (CAIX) and Glut1 (53). Tumors in both treatment conditions displayed little CAIX positivity. While control-treated tumors displayed remarkable Glut1 positivity, specifically in necrotic tumor areas (**Figure 7E**), lucanthone-treated tumors displayed minimal Glut1 positivity (**Figure 7F**). Quantification of Glut1 intensities is shown in **Figure 7G**. Glut1 expression in control tumors also mirrors expression patterns observed clinically (**Figure 7H**). These data illustrate that in addition to tumor-cell specific effects, lucanthone may modulate additional parameters of the tumor microenvironment. While Glut1 was reduced throughout the tumor, we observed that another glucose transporter, Glut4, was expressed throughout the tumor in saline- and lucanthone-treated conditions (**Figure S6**), suggesting that glucose transporter expression is not globally affected. As well, we observed an increase in the amount of cytotoxic T cells in the center of tumors in mice treated with lucanthone, which suggests that there may be a relief in the immunosuppressive nature fostered by gliomas after treatment (**Figure 7I**).

Targeting lysosomes is thought to exert effects on multiple cell types in the glioma microenvironment, potentially including GAM. Therefore, we assessed for differences in myeloid cell populations by staining for P2RY12 and TMEM119. In accordance with our previous work (32), P2RY12+ cells appeared mainly around the rim of gliomas in both treatment conditions (**Figure S7A**). However, we detected TMEM119+ cells throughout control-treated tumors and to a lesser extent in lucanthone-treated tumors (**Figures S7B, C**).

## Discussion

The pursuit of superior therapeutics for the treatment of high-grade glioma is limited in large part by the existence of the blood-brain barrier, which has evolved to exclude large and charged molecules from accumulating in the CNS at meaningful concentrations. Although substantial research has been conducted over the past several years to identify novel targets for targeting glioma without classical side effects associated with genotoxic stressors, failure of novel and repurposed drugs to reach the brain may limit their clinical use, even if they exert therapeutic effects in *in vivo* models of peripheral tumor (54). Additionally, the presence of GSC with their marked resistance to standard therapies, such as radiation and TMZ treatment, contribute to the inevitable recurrence and dismal prognosis of this disease (43, 44).

Our data show that lucanthone, a drug utilized for the treatment of schistosomal infections, targets autophagy in glioma cells, when administered systemically, and slows the growth of intracranial gliomas *in vivo*. These data, in addition to prior reports detailing its pharmacokinetic distribution in murine models (26), suggest that lucanthone may be able to enter the brain to act either as a monotherapy or work in concert with existing therapies.

Most interventions tailored to treating high-grade gliomas minimally prolong patient survival. Extensive research into the treatment resistance to TMZ, radiation, angiogenesis inhibitors, and tumor-treating fields therapy all point to the induction of cytoprotective autophagy as a means for treatment resistance and eventual disease progression (9–11, 45, 55–58). Oftentimes, chloroquine or hydroxychloroquine have been used as autophagy inhibitors in the pre-clinical setting and have been trialed in a myriad of different cancers. With specific respect to glioma, chloroquine exhibits poor penetration of the blood-brain barrier (59) and low potency (27), which may explain its lack of clinical efficacy. Lucanthone is a more potent autophagy inhibitor, and is well tolerated in the clinical setting. Additionally, our data show that at sub-cytotoxic concentrations, lucanthone may still be useful to augment the efficacy of TMZ (**Figure 2**). Future studies are warranted to detail its interaction with therapies such as radiation, angiogenesis inhibitors and tumor-treating fields *in vitro* and *in vivo*.

Lucanthone has been shown to act as a topoisomerase II poison as well as an APE1 inhibitor at high concentrations. Our results, however, advocate that its primary function would be the disruption of autophagy. After treatment, we observed extensive accumulation of autophagosomes in both KR158 and GL261 cells, also demonstrating that lucanthone exerts its effects independent of driver mutations. It is of particular interest that when glioma cells were cultured in stemness-promoting conditions, they exhibited increased sensitivity to lucanthone at doses as low as 3 μM. Since GSC are notoriously resistant to standard treatments, the development of adjuvant therapies that target a resistant sub-population may be useful in managing this disease and preventing recurrence. It is possible that lucanthone preferentially targets this sub-population by inducing lysosomal membrane permeabilization (LMP). Our data demonstrate that after lucanthone treatment, Cathepsin D is found throughout the cell, which may be due to lysosomal rupture and spilling of lysosomal contents into the cytoplasm. Prior reports have shown that GSC are susceptible to LMP (60–62), providing further evidence that interfering with lysosomal function may properly target cells spared from standard glioma treatments. We show here that lucanthone targeted glioma cells CD133^+^ glioma cells that have acquired resistance to TMZ, recapitulating previous reports that temozolomide induces glioma cells to acquire more stem-like characteristics (47). As there are no therapies currently approved for the treatment of recurrent glioblastoma, it would be of interest to develop a robust pipeline in which drugs are tested against glioma cells with an acquired resistance to temozolomide ± ionizing radiation.

To mechanistically explain lucanthone’s inhibitory effect on stemness, we probed for changes in LC3 and the stemness markers nestin, SOX2 and Olig2. We expected to observe increases in LC3 intensity in lucanthone-treated spheroid cultures. It should be noted that there were noticeable numbers of autophagosomes in control-treated spheroid cultures, strengthening the notion that GSC are more reliant on autophagy for survival at baseline conditions. However surprisingly there was a significant reduction in the number of cells in spheroids that stained positive for Olig2. In triple-negative breast tumor cells with constitutively active STAT3, the autophagy inhibitor chloroquine reduces active STAT3 (63). In glioma, inhibiting STAT3 activation by pharmacological or genetic means has been shown to reduce Olig2 levels (64), observations that may tie together lucanthone’s mechanism with the observed reduction in Olig2. These *in vitro* results were recapitulated *in vivo*: Tumors derived from control-treated mice exhibited robust Olig2 intensity, especially at the tumor border. Lucanthone reduced Olig2 levels at the border and core of the tumors (**Figure 6**). Olig2^+^ glioma cells exhibit increased resistance to standard therapies (65, 66), further encouraging the concomitant use of lucanthone with aforementioned interventions.

Gliomas exhibit dysregulated angiogenesis, which may contribute to the development of tumor hypoxia. Chloroquine was previously shown to act on endothelial cells in the melanoma tumor microenvironment. Chloroquine decreased the degradation of endothelial Notch 1, which functions to normalize tumor blood vessels and increases perfusion of the tumor. Herein, we find that the blood vessels of tumors treated with lucanthone exhibited increased circularity and reduced tortuosity. Decreasing tumor hypoxia may serve multiple functions, including increasing the delivery of systemic therapies to the whole tumor mass. In addition, eliminating pockets of hypoxia in gliomas through proper vessel perfusion could increase the efficacy of radiation therapy (67, 68) and restore the activity of cytotoxic T cells (69).

The advent of immunotherapies in the clinical setting has sparked an interest in understanding the role of both the innate and adaptive immune systems in the progression of aggressive tumor types, such as high-grade gliomas. Gliomas are comprised of multiple cell types specific to the CNS, and are heavily composed of CNS-resident microglia and blood-derived macrophages (70). Offsetting the tumor-promoting functions of these cells may directly slow the growth of gliomas and interact favorably with TMZ (49, 71, 72) and radiation (73). Investigations in peripheral tumor types, such as melanoma and hepatocellular carcinoma, revealed that late-stage autophagy inhibition with chloroquine, which was shown to act as an inhibitor of palmitoyl-protein thioesterase 1 (Ppt1) (74–76), reverses the immunosuppressive nature of tumor-associated macrophages and thus increases the efficacy of T-cell targeted PD-1 therapies (28, 76). While we have not yet identified the direct protein target of lucanthone action that results in autophagy inhibition, we hypothesize that, due to the structural similarity between lucanthone and chloroquine, Ppt1 may be an additional interactor, along with TopII and Ape1. Given that lucanthone may augment T cell infiltration into the glioma microenvironment (**Figure 7I**), future research may examine the extent to which lucanthone modulates the pro-/anti-tumorigenic function of glioma-associated microglia and macrophages alone and in combination with targeted therapies such as PD-1 inhibitors or radiation.

Taken together, our data support the concept that lucanthone may represent a sorely needed therapy to treat (recurrent/TMZ-resistant) high-grade gliomas. It may favorably interact with existing therapies through its direct effects on glioma cells, and may enhance therapeutic efficacy by modulating the function of endothelial cells and glioma stem cells. Exploring combinations of lucanthone with DNA-damaging therapies and immune-stimulating therapies may yield synergistic effects and improve our ability to clinically manage this intractable disease.

## Data Availability Statement

The original contributions presented in the study are included in the article/**Supplementary Material**. Further inquiries can be directed to the corresponding author.

## Ethics Statement

The animal study was reviewed and approved by IACUC Stony Brook University.

## Author Contributions

DR designed, performed and analyzed experiments and wrote drafts of the manuscript. GS, VM, and AW performed and analyzed experiments. RB provided lucanthone and assisted with experimental design and manuscript preparation. ST designed, supervised and analyzed experimental data, and edited manuscript drafts. All authors contributed to the article and approved the submitted version.

## Funding

This work was partially supported by an NIH F30CA257677 (DR), NIH T32GM008444 (DR) and a Stony Brook University (SBU) OVPR Seed Grant and SBU Bridge funds (ST).

## Conflict of Interest

The authors declare that the research was conducted in the absence of any commercial or financial relationships that could be construed as a potential conflict of interest.

## Publisher’s Note

All claims expressed in this article are solely those of the authors and do not necessarily represent those of their affiliated organizations, or those of the publisher, the editors and the reviewers. Any product that may be evaluated in this article, or claim that may be made by its manufacturer, is not guaranteed or endorsed by the publisher.

## References

[1] Omuro A DeAngelis LM. Glioblastoma and Other Malignant Gliomas: A Clinical Review. JAMA (2013) 310(17):1842–50. doi: 10.1001/jama.2013.280319 24193082

[2] Stupp R Taillibert S Kanner A Read W Steinberg D Lhermitte B. Effect of Tumor-Treating Fields Plus Maintenance Temozolomide vs Maintenance Temozolomide Alone on Survival in Patients With Glioblastoma: A Randomized Clinical Trial. JAMA (2017) 318(23):2306–16. doi: 10.1001/jama.2017.18718 PMC5820703 29260225

[3] Miyauchi JT Chen D Choi M Nissen JC Shroyer KR Djordevic S. Ablation of Neuropilin 1 From Glioma-Associated Microglia and Macrophages Slows Tumor Progression. Oncotarget (2016) 7(9):9801–14. doi: 10.18632/oncotarget.6877 PMC4891085 26755653

[4] Miyauchi JT Caponegro MD Chen D Choi MK Li M Tsirka SE. Deletion of Neuropilin 1 From Microglia or Bone Marrow-Derived Macrophages Slows Glioma Progression. Cancer Res (2018) 78(3):685–94. doi: 10.1158/0008-5472.CAN-17-1435 PMC5887044 29097606

[5] Valdor R Garcia-Bernal D Riquelme D Martinez CM Moraleda JM Cuervo AM. Glioblastoma Ablates Pericytes Antitumor Immune Function Through Aberrant Up-Regulation of Chaperone-Mediated Autophagy. Proc Natl Acad Sci USA (2019) 116(41):20655–65. doi: 10.1073/pnas.1903542116 PMC6789971 31548426

[6] Pyonteck SM Akkari L Schuhmacher AJ Bowman RL Sevenich L Quail DF. CSF-1R Inhibition Alters Macrophage Polarization and Blocks Glioma Progression. Nat Med (2013) 19(10):1264–72. doi: 10.1038/nm.3337 PMC3840724 24056773

[7] Chang AL Miska J Wainwright DA Dey M Rivetta CV Yu D. CCL2 Produced by the Glioma Microenvironment Is Essential for the Recruitment of Regulatory T Cells and Myeloid-Derived Suppressor Cells. Cancer Res (2016) 76(19):5671–82. doi: 10.1158/0008-5472.CAN-16-0144 PMC5050119 27530322

[8] Kanzawa T Germano IM Komata T Ito H Kondo Y Kondo S. Role of Autophagy in Temozolomide-Induced Cytotoxicity for Malignant Glioma Cells. Cell Death Differ (2004) 11(4):448–57. doi: 10.1038/sj.cdd.4401359 14713959

[9] Hori YS Hosoda R Akiyama Y Sebori R Wanibuchi M Mikami T. Chloroquine Potentiates Temozolomide Cytotoxicity by Inhibiting Mitochondrial Autophagy in Glioma Cells. J Neurooncol (2015) 122(1):11–20. doi: 10.1007/s11060-014-1686-9 25528635

[10] Ye H Chen M Cao F Huang H Zhan R Zheng X. Chloroquine, an Autophagy Inhibitor, Potentiates the Radiosensitivity of Glioma Initiating Cells by Inhibiting Autophagy and Activating Apoptosis. BMC Neurol (2016) 16(1):178. doi: 10.1186/s12883-016-0700-6 27644442 PMC5029068

[11] Abdul Rahim SA Dirkse A Oudin A Schuster A Bohler J Barthelemy V. Regulation of Hypoxia-Induced Autophagy in Glioblastoma Involves ATG9A. Br J Cancer (2017) 117(6):813–25. doi: 10.1038/bjc.2017.263 PMC5590001 28797031

[12] Xu J Zhang J Zhang Z Gao Z Qi Y Qiu W. Hypoxic Glioma-Derived Exosomes Promote M2-Like Macrophage Polarization by Enhancing Autophagy Induction. Cell Death Dis (2021) 12(4):373. doi: 10.1038/s41419-021-03664-1 33828078 PMC8026615

[13] Liang X De Vera ME Buchser WJ Romo de Vivar Chavez A Loughran P Beer Stolz D. Inhibiting Systemic Autophagy During Interleukin 2 Immunotherapy Promotes Long-Term Tumor Regression. Cancer Res (2012) 72(11):2791–801. doi: 10.1158/0008-5472.CAN-12-0320 PMC3417121 22472122

[14] DeVorkin L Pavey N Carleton G Comber A Ho C Lim J. Autophagy Regulation of Metabolism Is Required for CD8(+) T Cell Anti-Tumor Immunity. Cell Rep (2019) 27(2):502–513 e505. doi: 10.1016/j.celrep.2019.03.037 30970253

[15] Blair DM Hawking F Meeser CV Ross WF. Miracil; Clinical Trial on Patients Infected With Schistosoma Haematobium and S. Mansoni. Br J Pharmacol Chemother (1949) 4(1):68–80. doi: 10.1111/j.1476-5381.1949.tb00517.x 18124427 PMC1509891

[16] Alves W. Further Studies on the Treatment of Urinary Bilharziasis With Lucanthone Hydrochloride. Bull World Health Organ (1958) 18(5-6):1109–11. PMC2537957 13573140

[17] Berberian DA Freele H Rosi D Dennis EW Archer S. Schistosomicidal Activity of Lucanthone Hydrochloride, Hycanthone and Their Metabolites in Mice and Hamsters. J Parasitol (1967) 53(2):306–11. 6022391

[18] Archer S. Recent Progress in the Chemotherapy of Schistosomiasis. Prog Drug Res (1974) 18:15–24. doi: 10.1007/978-3-0348-7087-0_3 4616261

[19] Archer S Miller KJ Rej R Periana C Fricker L. Ring-Hydroxylated Analogues of Lucanthone as Antitumor Agents. J Med Chem (1982) 25(3):220–7. doi: 10.1021/jm00345a006 7069701

[20] Archer S Zayed AH Rej R Rugino TA. Analogues of Hycanthone and Lucanthone as Antitumor Agents. J Med Chem (1983) 26(9):1240–6. doi: 10.1021/jm00363a007 6887199

[21] Bases RE Mendez F. Topoisomerase Inhibition by Lucanthone, an Adjuvant in Radiation Therapy. Int J Radiat Oncol Biol Phys (1997) 37(5):1133–7. doi: 10.1016/s0360-3016(97)00113-2 9169823

[22] Dassonneville L Bailly C. Stimulation of Topoisomerase II-Mediated DNA Cleavage by an Indazole Analogue of Lucanthone. Biochem Pharmacol (1999) 58(8):1307–12. doi: 10.1016/s0006-2952(99)00221-x 10487533

[23] Luo M Kelley MR. Inhibition of the Human Apurinic/Apyrimidinic Endonuclease (APE1) Repair Activity and Sensitization of Breast Cancer Cells to DNA Alkylating Agents With Lucanthone. Anticancer Res (2004) 24(4):2127–34. 15330152

[24] Naidu MD Agarwal R Pena LA Cunha L Mezei M Shen M. Lucanthone and Its Derivative Hycanthone Inhibit Apurinic Endonuclease-1 (APE1) by Direct Protein Binding. PloS One (2011) 6(9):e23679. doi: 10.1371/journal.pone.0023679 21935361 PMC3174134

[25] Turner S Bases R Pearlman A Nobler M Kabakow B. The Adjuvant Effect of Lucanthone (Miracil D) in Clinical Radiation Therapy. Radiology (1975) 114(3):729–31. doi: 10.1148/114.3.729 1118579

[26] Del Rowe JD Bello J Mitnick R Sood B Filippi C Moran J. Accelerated Regression of Brain Metastases in Patients Receiving Whole Brain Radiation and the Topoisomerase II Inhibitor, Lucanthone. Int J Radiat Oncol Biol Phys (1999) 43(1):89–93. doi: 10.1016/s0360-3016(98)00374-5 9989518

[27] Carew JS Espitia CM Esquivel JA 2nd Mahalingam D Kelly KR Reddy G. Lucanthone Is a Novel Inhibitor of Autophagy That Induces Cathepsin D-Mediated Apoptosis. J Biol Chem (2011) 286(8):6602–13. doi: 10.1074/jbc.M110.151324 PMC3057822 21148553

[28] Chen D Xie J Fiskesund R Dong W Liang X Lv J. Chloroquine Modulates Antitumor Immune Response by Resetting Tumor-Associated Macrophages Toward M1 Phenotype. Nat Commun (2018) 9(1):873. doi: 10.1038/s41467-018-03225-9 29491374 PMC5830447

[29] Ausman JI Shapiro WR Rall DP. Studies on the Chemotherapy of Experimental Brain Tumors: Development of an Experimental Model. Cancer Res (1970) 30(9):2394–400. 5475483

[30] Reilly KM Loisel DA Bronson RT McLaughlin ME Jacks T. Nf1;Trp53 Mutant Mice Develop Glioblastoma With Evidence of Strain-Specific Effects. Nat Genet (2000) 26(1):109–13. doi: 10.1038/79075 10973261

[31] Yi L Zhou C Wang B Chen T Xu M Xu L. Implantation of GL261 Neurospheres Into C57/BL6 Mice: A More Reliable Syngeneic Graft Model for Research on Glioma-Initiating Cells. Int J Oncol (2013) 43(2):477–84. doi: 10.3892/ijo.2013.1962 23708048

[32] Caponegro MD Oh K Madeira MM Radin D Sterge N Tayyab M. A Distinct Microglial Subset at the Tumor-Stroma Interface of Glioma. Glia (2021) 69(7):1767–81. doi: 10.1002/glia.23991 PMC8113099 33704822

[33] Zhai H Acharya S Gravanis I Mehmood S Seidman RJ Shroyer KR. Annexin A2 Promotes Glioma Cell Invasion and Tumor Progression. J Neurosci (2011) 31(40):14346–60. doi: 10.1523/JNEUROSCI.3299-11.2011 PMC3201988 21976520

[34] Kumar V Radin D Leonardi D. Probing the Oncolytic and Chemosensitizing Effects of Dihydrotanshinone in an *In Vitro* Glioblastoma Model. Anticancer Res (2017) 37(11):6025–30. doi: 10.21873/anticanres.12049 29061781

[35] Radin DP Purcell R Lippa AS. Oncolytic Properties of Ampakines *In Vitro* . Anticancer Res (2018) 38(1):265–9. doi: 10.21873/anticanres.12217 29277782

[36] Kumar V Radin D Leonardi D. Studies Examining the Synergy Between Dihydrotanshinone and Temozolomide Against MGMT+ Glioblastoma Cells *In Vitro*: Predicting Interactions With the Blood-Brain Barrier. BioMed Pharmacother (2019) 109:386–90. doi: 10.1016/j.biopha.2018.10.069 30399573

[37] Xu J Patel NH Saleh T Cudjoe EK Jr. Alotaibi M Wu Y. Differential Radiation Sensitivity in P53 Wild-Type and P53-Deficient Tumor Cells Associated With Senescence But Not Apoptosis or (Nonprotective) Autophagy. Radiat Res (2018) 190(5):538–57. doi: 10.1667/RR15099.1 PMC7141768 30132722

[38] Tamamori-Adachi M Koga A Susa T Fujii H Tsuchiya M Okinaga H. DNA Damage Response Induced by Etoposide Promotes Steroidogenesis *via* GADD45A in Cultured Adrenal Cells. Sci Rep (2018) 8(1):9636. doi: 10.1038/s41598-018-27938-5 29941883 PMC6018231

[39] Racoma IO Meisen WH Wang QE Kaur B Wani AA. Thymoquinone Inhibits Autophagy and Induces Cathepsin-Mediated, Caspase-Independent Cell Death in Glioblastoma Cells. PloS One (2013) 8(9):e72882. doi: 10.1371/journal.pone.0072882 24039814 PMC3767730

[40] Ferrer-Font L Villamanan L Arias-Ramos N Vilardell J Plana M Ruzzene M. Targeting Protein Kinase CK2: Evaluating CX-4945 Potential for GL261 Glioblastoma Therapy in Immunocompetent Mice. Pharmaceuticals (Basel) (2017) 10(1):24. doi: 10.3390/ph10010024 PMC5374428 28208677

[41] Roberts NB Alqazzaz A Hwang JR Qi X Keegan AD Kim AJ. Oxaliplatin Disrupts Pathological Features of Glioma Cells and Associated Macrophages Independent of Apoptosis Induction. J Neurooncol (2018) 140(3):497–507. doi: 10.1007/s11060-018-2979-1 30132163 PMC6580860

[42] Couturier CP Ayyadhury S Le PU Nadaf J Monlong J Riva G. Single-Cell RNA-Seq Reveals That Glioblastoma Recapitulates a Normal Neurodevelopmental Hierarchy. Nat Commun (2020 3406) 11(1):3406. doi: 10.1038/s41467-020-17186-5 32641768 PMC7343844

[43] Bao S Wu Q McLendon RE Hao Y Shi Q Hjelmeland AB. Glioma Stem Cells Promote Radioresistance by Preferential Activation of the DNA Damage Response. Nature (2006) 444(7120):756–60. doi: 10.1038/nature05236 17051156

[44] Chen J Li Y Yu TS McKay RM Burns DK Kernie SG. A Restricted Cell Population Propagates Glioblastoma Growth After Chemotherapy. Nature (2012) 488(7412):522–6. doi: 10.1038/nature11287 PMC3427400 22854781

[45] Buccarelli M Marconi M Pacioni S De Pascalis I D’Alessandris QG Martini M. Inhibition of Autophagy Increases Susceptibility of Glioblastoma Stem Cells to Temozolomide by Igniting Ferroptosis. Cell Death Dis (2018) 9(8):841. doi: 10.1038/s41419-018-0864-7 30082680 PMC6079099

[46] Abbas S Singh SK Saxena AK Tiwari S Sharma LK Tiwari M. Role of Autophagy in Regulation of Glioma Stem Cells Population During Therapeutic Stress. J Stem Cells Regener Med (2020) 16(2):80–9. doi: 10.46582/jsrm.1602012 PMC7772813 33414584

[47] Lee G Auffinger B Guo D Hasan T Deheeger M Tobias AL. Dedifferentiation of Glioma Cells to Glioma Stem-Like Cells By Therapeutic Stress-Induced HIF Signaling in the Recurrent GBM Model. Mol Cancer Ther (2016) 15(12):3064–76. doi: 10.1158/1535-7163.MCT-15-0675 PMC5298928 27765847

[48] Azambuja JH da Silveira EF de Carvalho TR Oliveira PS Pacheco S do Couto CT. Glioma Sensitive or Chemoresistant to Temozolomide Differentially Modulate Macrophage Protumor Activities. Biochim Biophys Acta Gen Subj (2017) 1861(11 Pt A):2652–62. doi: 10.1016/j.bbagen.2017.07.007 28713019

[49] Li J Sun Y Sun X Zhao X Ma Y Wang Y. AEG-1 Silencing Attenuates M2-Polarization of Glioma-Associated Microglia/Macrophages and Sensitizes Glioma Cells to Temozolomide. Sci Rep (2021) 11(1):17348. doi: 10.1038/s41598-021-96647-3 34462446 PMC8405821

[50] Bastola S Pavlyukov MS Yamashita D Ghosh S Cho H Kagaya N. Glioma-Initiating Cells at Tumor Edge Gain Signals From Tumor Core Cells to Promote Their Malignancy. Nat Commun (2020) 11(1):4660. doi: 10.1038/s41467-020-18189-y 32938908 PMC7494913

[51] Puchalski RB Shah N Miller J Dalley R Nomura SR Yoon JG. An Anatomic Transcriptional Atlas of Human Glioblastoma. Science (2018) 360(6389):660–3. doi: 10.1126/science.aaf2666 PMC6414061 29748285

[52] Maes H Kuchnio A Peric A Moens S Nys K De Bock K. Tumor Vessel Normalization by Chloroquine Independent of Autophagy. Cancer Cell (2014) 26(2):190–206. doi: 10.1016/j.ccr.2014.06.025 25117709

[53] Li Z Bao S Wu Q Wang H Eyler C Sathornsumetee S. Hypoxia-Inducible Factors Regulate Tumorigenic Capacity of Glioma Stem Cells. Cancer Cell (2009) 15(6):501–13. doi: 10.1016/j.ccr.2009.03.018 PMC2693960 19477429

[54] Mulkearns-Hubert EE Torre-Healy LA Silver DJ Eurich JT Bayik D Serbinowski E. Development of a Cx46 Targeting Strategy for Cancer Stem Cells. Cell Rep (2019) 27(4):1062–1072 e1065. doi: 10.1016/j.celrep.2019.03.079 31018124 PMC6497083

[55] Yan Y Xu Z Dai S Qian L Sun L Gong Z. Targeting Autophagy to Sensitive Glioma to Temozolomide Treatment. J Exp Clin Cancer Res (2016) 35:23. doi: 10.1186/s13046-016-0303-5 26830677 PMC4736617

[56] Shteingauz A Porat Y Voloshin T Schneiderman RS Munster M Zeevi E. AMPK-Dependent Autophagy Upregulation Serves as a Survival Mechanism in Response to Tumor Treating Fields (TTFields). Cell Death Dis (2018) 9(11):1074. doi: 10.1038/s41419-018-1085-9 30341282 PMC6195570

[57] Kim EH Jo Y Sai S Park MJ Kim JY Kim JS. Tumor-Treating Fields Induce Autophagy by Blocking the Akt2/miR29b Axis in Glioblastoma Cells. Oncogene (2019) 38(39):6630–46. doi: 10.1038/s41388-019-0882-7 31375748

[58] Shi J Dong X Li H Wang H Jiang Q Liu L. Nicardipine Sensitizes Temozolomide by Inhibiting Autophagy and Promoting Cell Apoptosis in Glioma Stem Cells. Aging (Albany NY) (2021) 13(5):6820–31. doi: 10.18632/aging.202539 PMC7993688 33621205

[59] Roy L Poirier M Fortin D. P11.39 Chloroquine as an Anti-Glioblastoma Therapeutic: Repurposing of an Old Drug. Neuro-Oncology (2019) 21(Supplement_3):iii51–2. doi: 10.1093/neuonc/noz126.185

[60] Le Joncour V Filppu P Hyvonen M Holopainen M Turunen SP Sihto H. Vulnerability of Invasive Glioblastoma Cells to Lysosomal Membrane Destabilization. EMBO Mol Med (2019) 11(6):e9034. doi: 10.15252/emmm.201809034 31068339 PMC6554674

[61] Jacobs KA Andre-Gregoire G Maghe C Thys A Li Y Harford-Wright E. Paracaspase MALT1 Regulates Glioma Cell Survival by Controlling Endo-Lysosome Homeostasis. EMBO J (2020) 39(1):e102030. doi: 10.15252/embj.2019102030 31774199 PMC6939194

[62] Zhou W Guo Y Zhang X Jiang Z. Lys05 Induces Lysosomal Membrane Permeabilization and Increases Radiosensitivity in Glioblastoma. J Cell Biochem (2020) 121(2):2027–37. doi: 10.1002/jcb.29437 31642111

[63] Maycotte P Gearheart CM Barnard R Aryal S Mulcahy Levy JM Fosmire SP. STAT3-Mediated Autophagy Dependence Identifies Subtypes of Breast Cancer Where Autophagy Inhibition Can Be Efficacious. Cancer Res (2014) 74(9):2579–90. doi: 10.1158/0008-5472.CAN-13-3470 PMC4008672 24590058

[64] Guryanova OA Wu Q Cheng L Lathia JD Huang Z Yang J. Nonreceptor Tyrosine Kinase BMX Maintains Self-Renewal and Tumorigenic Potential of Glioblastoma Stem Cells by Activating STAT3. Cancer Cell (2011) 19(4):498–511. doi: 10.1016/j.ccr.2011.03.004 21481791 PMC3076106

[65] Mehta S Huillard E Kesari S Maire CL Golebiowski D Harrington EP. The Central Nervous System-Restricted Transcription Factor Olig2 Opposes P53 Responses to Genotoxic Damage in Neural Progenitors and Malignant Glioma. Cancer Cell (2011) 19(3):359–71. doi: 10.1016/j.ccr.2011.01.035 PMC3070398 21397859

[66] Liu H Weng W Guo R Zhou J Xue J Zhong S. Olig2 SUMOylation Protects Against Genotoxic Damage Response by Antagonizing P53 Gene Targeting. Cell Death Differ (2020) 27(11):3146–61. doi: 10.1038/s41418-020-0569-1 PMC7560653 32483381

[67] Dings RP Loren M Heun H McNiel E Griffioen AW Mayo KH. Scheduling of Radiation With Angiogenesis Inhibitors Anginex and Avastin Improves Therapeutic Outcome *via* Vessel Normalization. Clin Cancer Res (2007) 13(11):3395–402. doi: 10.1158/1078-0432.CCR-06-2441 PMC2914684 17545548

[68] Sheehan J Cifarelli CP Dassoulas K Olson C Rainey J Han S. Trans-Sodium Crocetinate Enhancing Survival and Glioma Response on Magnetic Resonance Imaging to Radiation and Temozolomide. J Neurosurg (2010) 113(2):234–9. doi: 10.3171/2009.11.JNS091314 20001586

[69] Noman MZ Janji B Kaminska B Van Moer K Pierson S Przanowski P. Blocking Hypoxia-Induced Autophagy in Tumors Restores Cytotoxic T-Cell Activity and Promotes Regression. Cancer Res (2011) 71(18):5976–86. doi: 10.1158/0008-5472.CAN-11-1094 21810913

[70] Radin DP Tsirka SE. Interactions Between Tumor Cells, Neurons, and Microglia in the Glioma Microenvironment. Int J Mol Sci (2020) 21(22):8476. doi: 10.3390/ijms21228476 PMC7698134 33187183

[71] Chuang HY Su YK Liu HW Chen CH Chiu SC Cho DY. Preclinical Evidence of STAT3 Inhibitor Pacritinib Overcoming Temozolomide Resistance *via* Downregulating miR-21-Enriched Exosomes From M2 Glioblastoma-Associated Macrophages. J Clin Med (2019) 8(7):959. doi: 10.3390/jcm8070959 PMC6678764 31269723

[72] Li J Kaneda MM Ma J Li M Shepard RM Patel K. PI3Kgamma Inhibition Suppresses Microglia/TAM Accumulation in Glioblastoma Microenvironment to Promote Exceptional Temozolomide Response. Proc Natl Acad Sci USA (2021) 118(16):e2009290118. doi: 10.1073/pnas.2009290118 33846242 PMC8072253

[73] Akkari L Bowman RL Tessier J Klemm F Handgraaf SM de Groot M. Dynamic Changes in Glioma Macrophage Populations After Radiotherapy Reveal CSF-1R Inhibition as a Strategy to Overcome Resistance. Sci Transl Med (2020) 12(552):eaaw7843 . doi: 10.1126/scitranslmed.aaw7843 32669424

[74] Nicastri MC Rebecca VW Amaravadi RK Winkler JD. Dimeric Quinacrines as Chemical Tools to Identify PPT1, a New Regulator of Autophagy in Cancer Cells. Mol Cell Oncol (2018) 5(1):e1395504. doi: 10.1080/23723556.2017.1395504 29404393 PMC5791860

[75] Rebecca VW Nicastri MC Fennelly C Chude CI Barber-Rotenberg JS Ronghe A. PPT1 Promotes Tumor Growth and Is the Molecular Target of Chloroquine Derivatives in Cancer. Cancer Discov (2019) 9(2):220–9. doi: 10.1158/2159-8290.CD-18-0706 PMC6368875 30442709

[76] Sharma G Ojha R Noguera-Ortega E Rebecca VW Attanasio J Liu S. PPT1 Inhibition Enhances the Antitumor Activity of Anti-PD-1 Antibody in Melanoma. JCI Insight (2020) 5(17):e133225. doi: 10.1172/jci.insight.133225 PMC7526447 32780726

