# Supplementary material for: Lucanthone Targets Lysosomes to Perturb Glioma Proliferation, Chemoresistance and Stemness, and Slows Tumor Growth In Vivo
Source: Front Oncol. 2022 Apr 14;12:852940. doi: 10.3389/fonc.2022.852940 (PMC9048484; doi:10.3389/fonc.2022.852940)
Supplement: Supplementary file 1 [file DataSheet_1.pdf]

Supplementary Figures

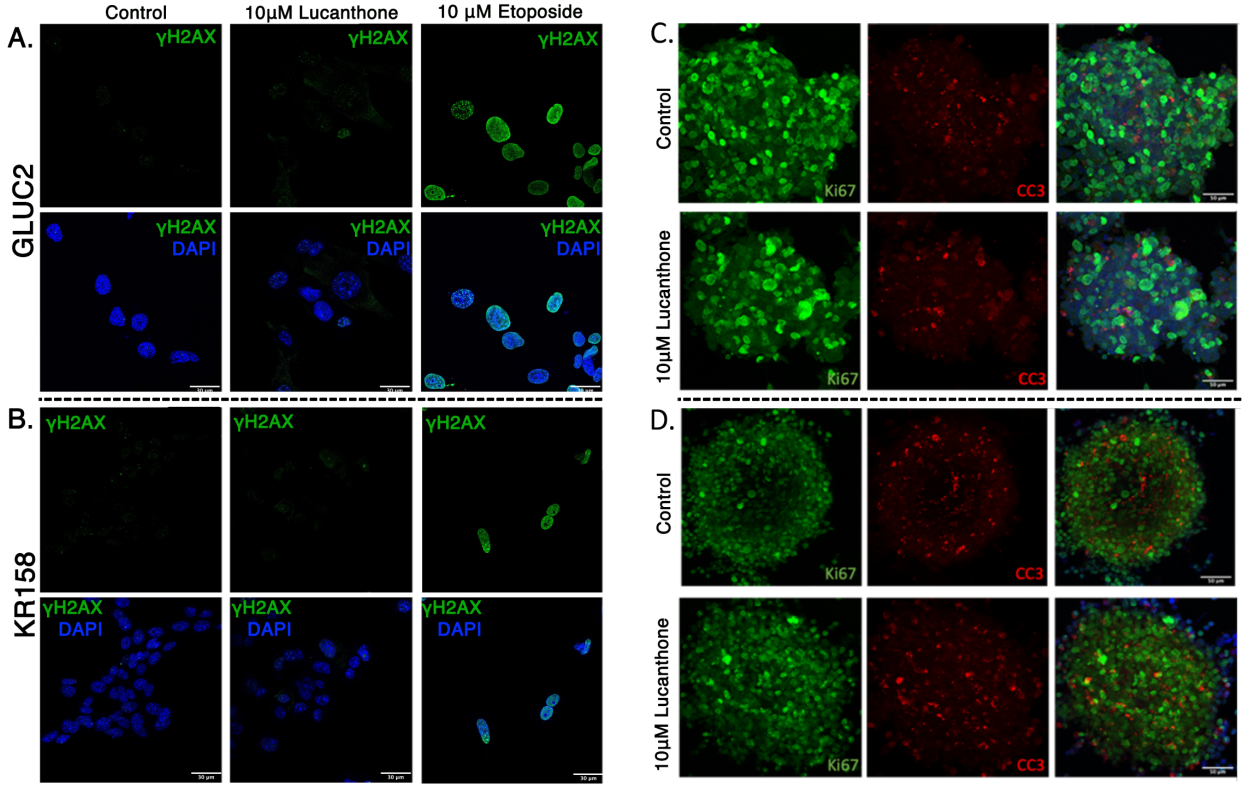

Figure S1. Effects of Lucanthone on γH2AX. GLUC2 (**A**) and KR158 cells (**B**) were treated with lucanthone or the topoisomerase 2 inhibitor etoposide for 48 hours, after which cells were stained for γH2AX. Representative photomicrographs of Ki67 and cleaved caspase 3 (CC3) in GLUC2 (**C**) and KR158 (**D**) spheroids treated with control and 10μM Lucanthone for 48 hours.

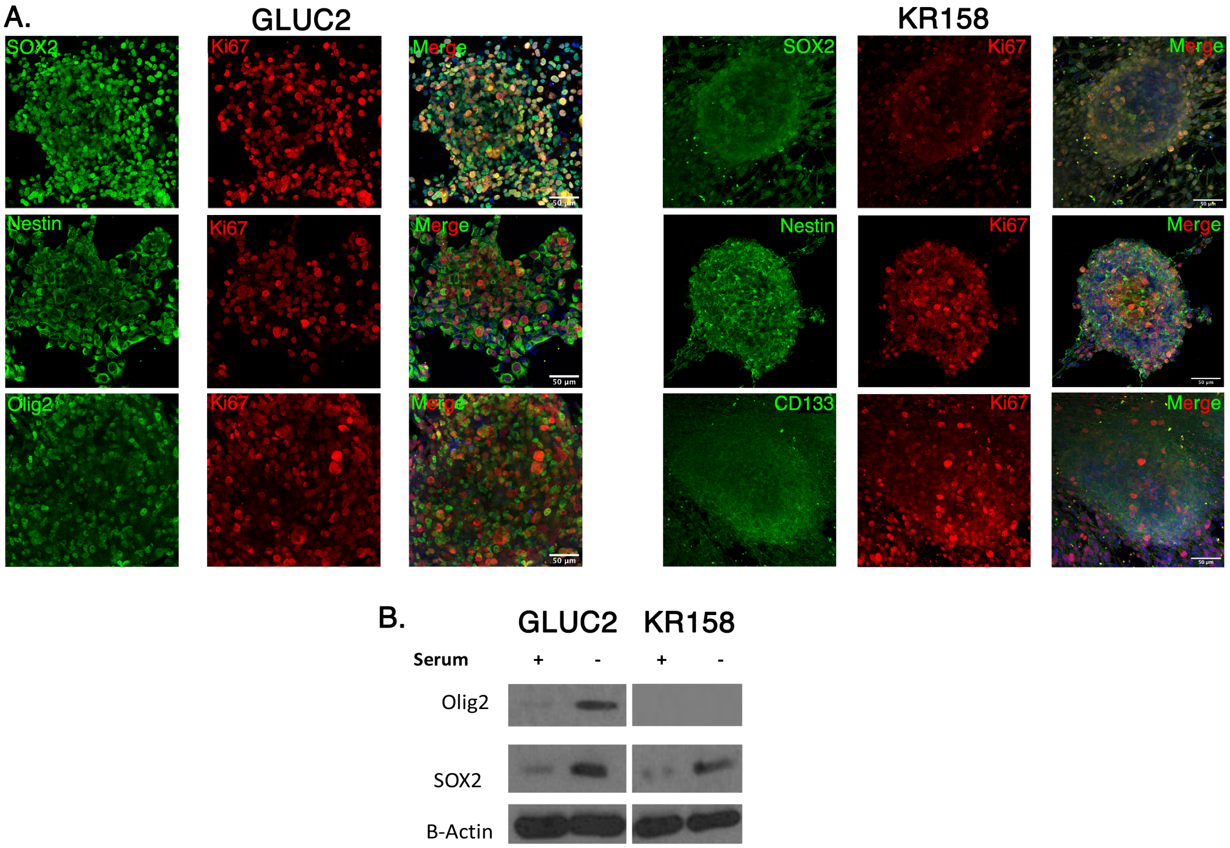

Figure S2. **Glioma spheroids express stemness markers.** A) GLUC2 and KR158 spheroids were allowed for 24hrs to adhere to glass slides that had been pre-coated with Geltrex for an hour. The spheroids were then PFA-fixed and stained for SOX2, nestin, Olig2, CD133 and Ki67. GLUC2 spheroids expressed SOX2, nestin, Olig2 were also positive for the proliferation marker Ki67. KR158 spheroids expressed SOX2, CD133 and nestin, in addition to Ki67. Results are representative of 3 independent experiments; B) Spheroids also express increased levels of SOX2 and Olig2, as measured by western blot.

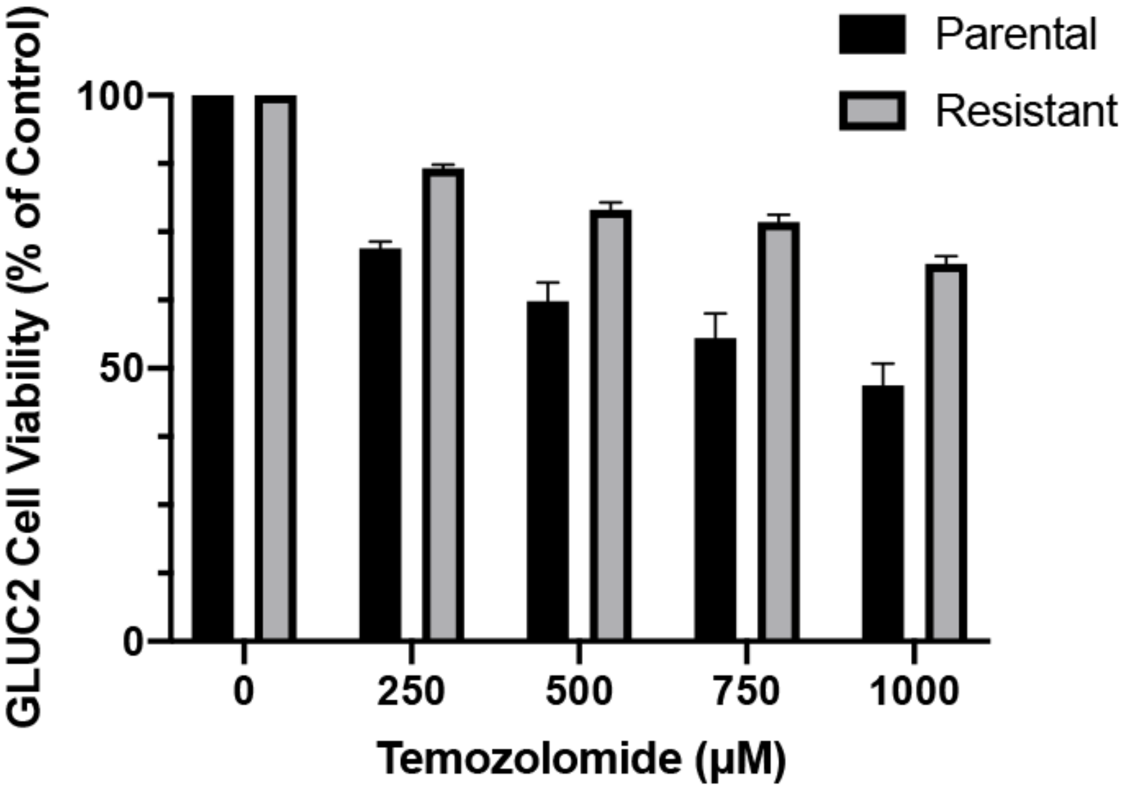

Figure S3. Parental GLUC2 cells are more sensitive to TMZ. GLUC2 cells (parental) as well as cells that had developed resistance to TMZ were exposed to increasing concentrations of TMZ. The culture viability was examined 72 hours after treatment by MTT. Bars are mean +/- SEM, n=4 independent experiments. P<0.01, Two-way ANOVA, indicating significant effects by dose and between cell lines.

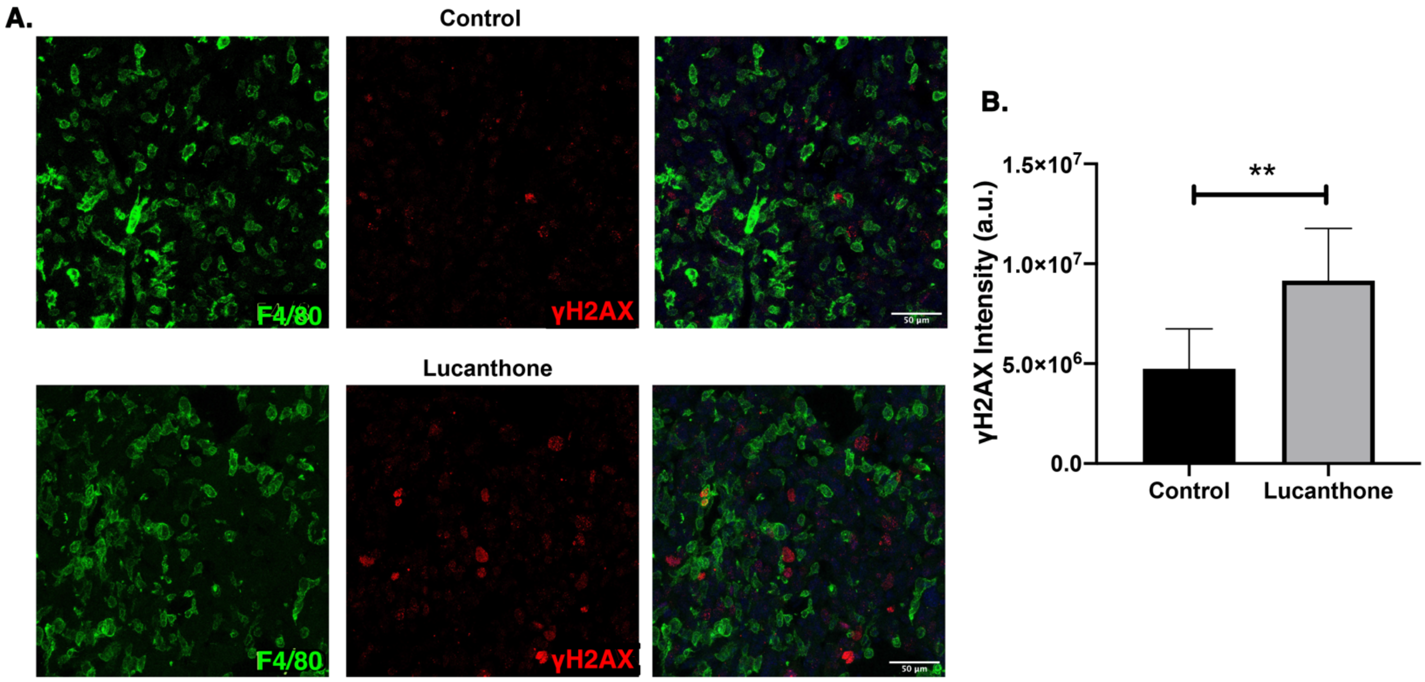

Figure S4. Lucanthone increases γH2AX in tumors in vivo. Representative micrographs of tumor sections stained for the GAM marker F4/80 and γH2AX. Quantification of 7 animals per group. Bars are mean +/- SEM. **$p<0.01$, t-test.

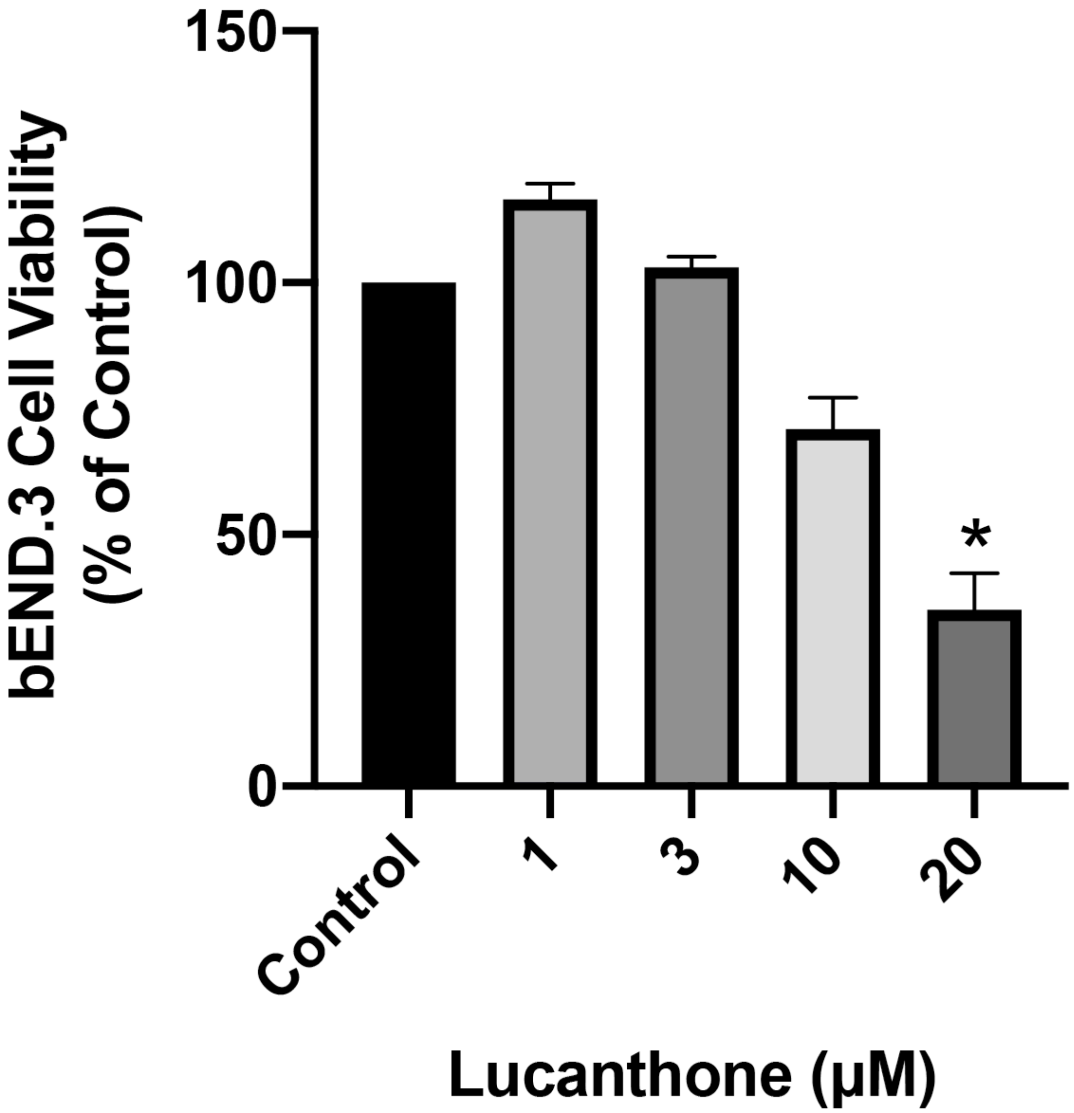

Figure S5. Lucanthone compromises bEND.3 cell viability at high concentrations only. bEND.3 cells were treated with lucanthone for 72 hours and then an MTT assay was performed. Data are mean +/- SEM of 3 independent experiments. ANOVA, $p<0.01$, demonstrating a significant dose-response effect. *$p<0.05$, Sidak multiple comparison test.

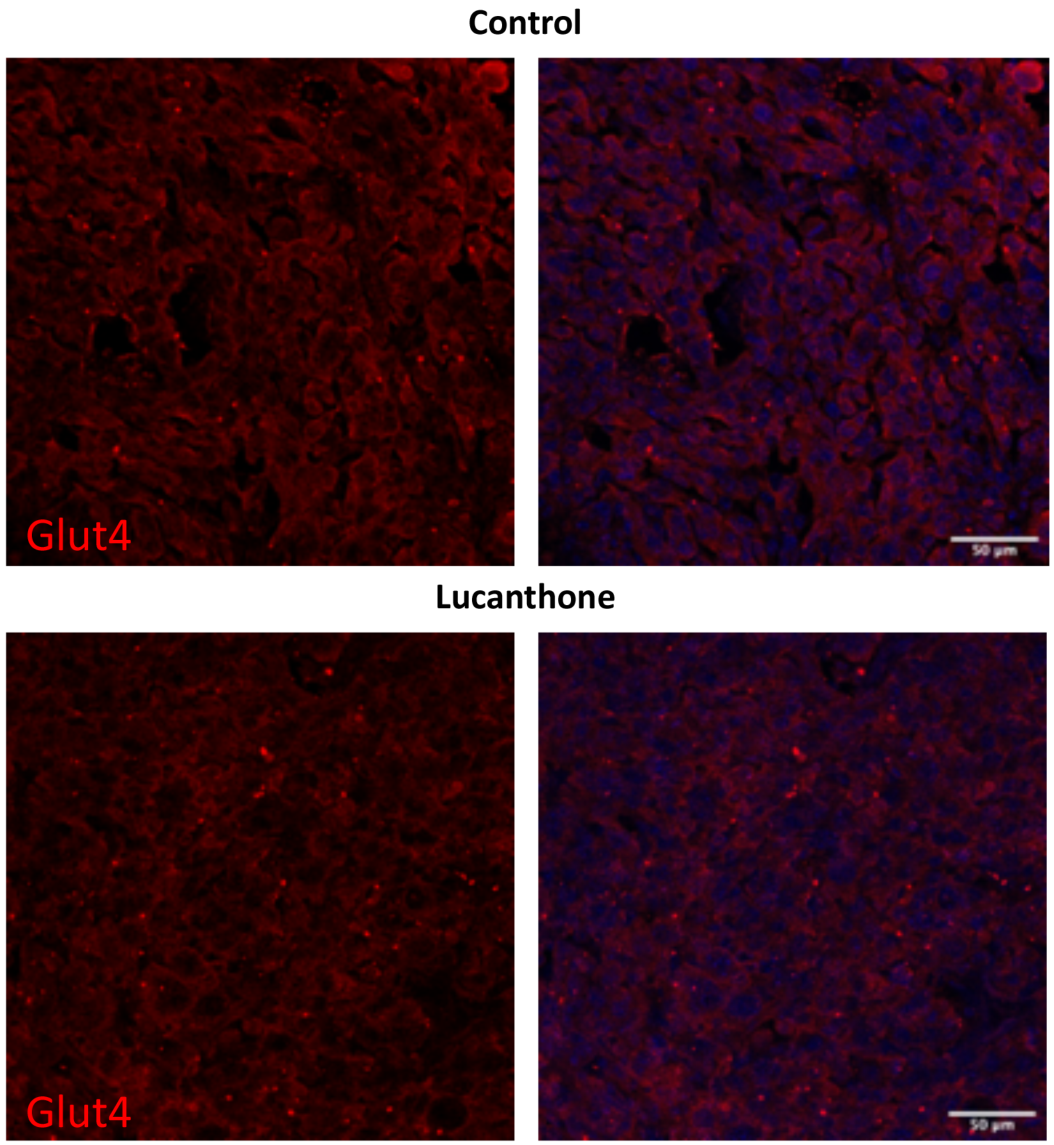

Figure S6. The levels of Glut4 are not affected by lucanthone treatment in vivo. Control- and lucanthone-treated tumors were stained for Glut4. Levels were similar among treatment groups.

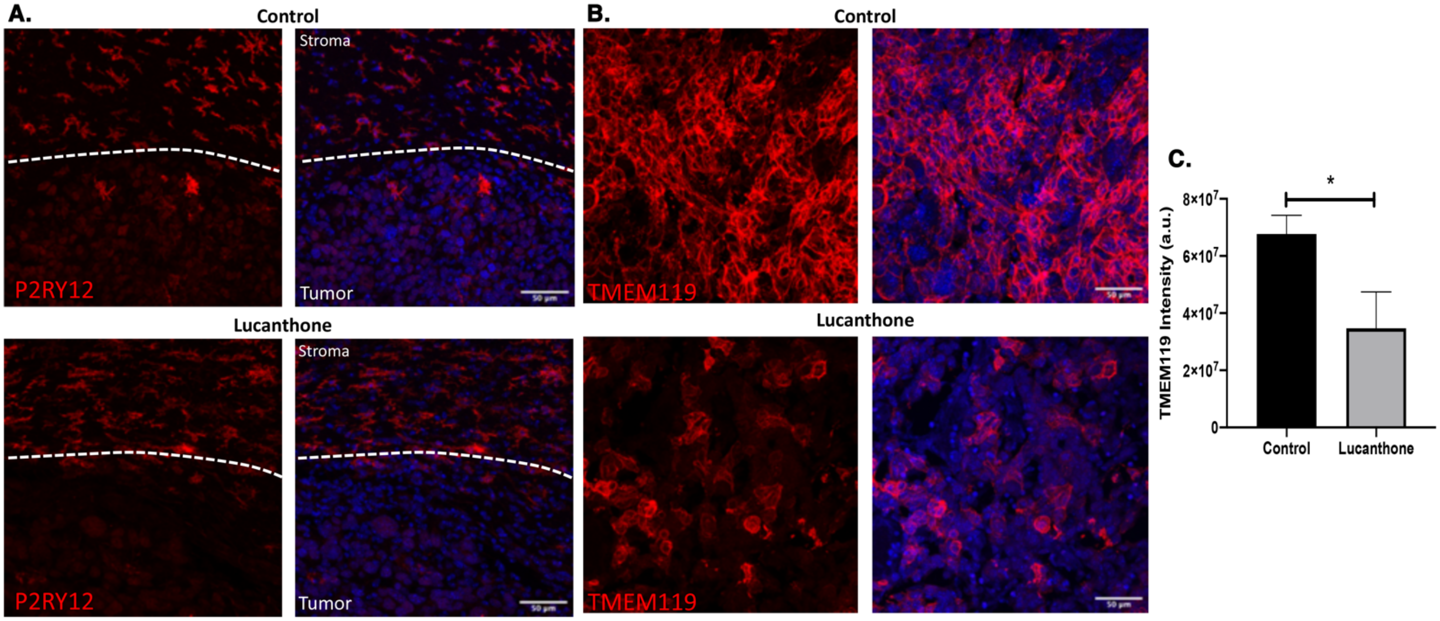

Figure S7. Effects of lucanthone on P2RY12 and TMEM119 levels in vivo. A) Control- and lucanthone-treated tumors were stained for P2RY12. Levels were similar among treatment groups; **B)** Representative micrographs of tumor core sections stained for TMEM119; **C)** Quantification of 5 animals per group. Bars are mean +/- SEM. *$p<0.01$, t-test.
